# ROLE OF STATINS IN CIRRHOTIC PORTAL HYPERTENSION: META-ANALYSIS OF RANDOMIZED STUDIES

**DOI:** 10.1590/S0004-2803.24612025-036

**Published:** 2026-05-18

**Authors:** Mariana Oliveira Amarante MORENO, Cláudio Luiz da Silva Lima PAZ, Mariana Alves Nascimento RODRIGUES, Antonio Carlos Dias ANDRADE, Maria Gabriela Fernandes DEZAN, Lourianne Nascimento CAVALCANTE, André Castro LYRA

**Affiliations:** 1 Escola Bahiana de Medicina e Saúde Pública, Salvador, Bahia, Brasil.; 2 Universidade Federal da Bahia, Programa de Pós-Graduação em Medicina e Saúde (PPgMS), Salvador, Bahia, Brasil.; 3 Universidade Federal do Vale do São Francisco, Petrolina, Pernambuco, Brasil.; 4 Universidade Federal da Bahia, Hospital Universitário Professor Edgard Santos, Serviço de Gastro-Hepatologia, Salvador, Bahia, Brasil.; 5 Instituto Do’r de Ensino e Pesquisa / Hospital São Rafael, Serviço de Gastro-Hepatologia, Salvador, BA, Brasil.

**Keywords:** Liver, cirrhosis, statin, survival, portal hypertension, meta-analysis., Fígado, cirrose, estatina, sobrevida, hipertensão portal, metanálise

## Abstract

**Background:**

Physicians often use caution when prescribing statins to patients with chronic liver disease (CLD) due to potential hepatotoxicity. However, recent evidence indicates that hepatotoxicity is uncommon, and the risks of not using statins may often outweigh those associated with their use. Additionally, recent findings suggest that statins may have clinically beneficial effects on cirrhosis due to their pleiotropic properties.

**Objective:**

To evaluate the impact of statin use on the number of patients with chronic liver disease alive at the end of the trials and to assess its effects on portal hypertension, ascites, hepatic encephalopathy, variceal hemorrhage, and hepatocellular carcinoma.

**Methods:**

This meta-analysis analyzed 9 randomized clinical trials published in the MEDLINE, EMBASE, and SCOPUS databases that evaluated the use of statins in patients with cirrhosis. These studies included a total of 811 subjects with portal hypertension, of which 401 patients were in the intervention group. We assessed risk of bias using the Risk of Bias 2 (RoB 2) tool for randomized clinical trials, and we analyzed the quality of evidence using the GRADE (Grading of Recommendations Assessment, Development, and Evaluation) scale. We extracted data on the number of events and the total number of patients to perform a meta-analysis of proportions using the R software with the “meta” package (version 4.9-6).

**Results:**

Our meta-analysis on patients alive at the end of the trials included 5 studies, involving a total of 718 patients (352 in the statin group and 366 in the control group). From these, 618 patients were alive at the end of the studies, with 301 from the intervention group and 287 from the control group. The overall odds ratio (OR) between the groups was 1.79 (95%CI -1.04 to 3.06; I²: 20.3%), favoring the use of statins. The results for variceal gastrointestinal bleeding showed an OR of 0.67 (95%CI, 0.41 to 1.09; I²: 0%); for hemodynamic response, the OR was 2.41 (95%CI, 0.65 to 8.92; I²: 66%); for hepatic encephalopathy, the OR was 0.41 (95%CI, 0.06 to 2.77; I²: 61%); for myalgia, an OR of 0.74 (95%CI, 0.22 to 2.46; I²: 1%); and for ascites, the OR was 0.84 (95%CI, 0.48 to 1.48; I²: 0%).

**Conclusion:**

The use of statins is associated with a greater number of patients with hepatic cirrhosis alive at the end of the trials. However, we were unable to determine the reason for this potential beneficial effect.

## HIGHLIGHTS

• Statins are often underused in chronic liver disease due to concerns about hepatotoxicity, despite evidence showing such toxicity is uncommon.

• Growing data suggest statins may provide clinical benefits in cirrhosis through pleiotropic effects.

• This meta-analysis included 9 randomized clinical trials with 811 patients with portal hypertension (401 receiving statins). Statin therapy was associated with more patients alive at the end of the trials compared to the control group.

• Statins did not show benefit in reducing variceal gastrointestinal bleeding, hepatic encephalopathy, ascites, myalgia, or hemodynamic response.

## RESUMO

**Contexto:**

Médicos frequentemente utilizam precaução ao prescrever estatinas para pacientes com doença hepática crônica (DHC) devido ao potencial de hepatotoxicidade. No entanto, evidências recentes indicam que a hepatotoxicidade é incomum, e os riscos de não utilizar estatinas frequentemente superam os riscos associados ao seu uso. Além disso, descobertas recentes sugerem que as estatinas podem ter efeitos clínicos benéficos na cirrose devido às suas propriedades pleiotrópicas.

**Objetivos:**

Avaliar o impacto do uso de estatinas na sobrevida de pacientes com doença hepática crônica e analisar seus efeitos sobre a hipertensão portal, ascite, encefalopatia hepática, hemorragia varicosa e carcinoma hepatocelular.

**Métodos:**

Esta metanalise analisou nove ensaios clínicos randomizados publicados nas bases de dados MEDLINE, EMBASE e SCOPUS que avaliaram o uso de estatinas em pacientes com cirrose. Esses estudos incluíram um total de 811 indivíduos com hipertensão portal, dos quais 401 pacientes estavam no grupo de intervenção. O risco de viés foi avaliado utilizando a ferramenta Risk of Bias 2 (RoB 2) para ensaios clínicos randomizados, e a qualidade das evidências foi analisada com a escala GRADE (Grading of Recommendations Assessment, Development, and Evaluation). Foram extraídos dados sobre o número de eventos e o total de pacientes avaliados para realizar meta-análises de proporções utilizando o software R com o pacote “meta” (versão 4.9-6).

**Resultados:**

Nossa meta-análise sobre sobrevida incluiu cinco estudos com um total de 718 pacientes (352 no grupo que usou estatinas e 366 no grupo controle). Desses, 618 pacientes estavam vivos ao final dos estudos, sendo 301 do grupo de intervenção e 287 do grupo controle. A razão de chances (odds ratio, OR) geral entre os grupos foi de **1.79** (95%CI -1.04 to 3.06; I²: 20.3%), favorecendo o uso de estatinas. Os resultados para sangramento gastrointestinal varicoso mostraram uma OR de **0,67** (IC95%: -0,41 a 1,09; I²: 0%); para resposta hemodinâmica, a OR foi de **2,41** (IC95%: -0,65 a 8,92; I²: 66%); para encefalopatia hepática, a OR foi de **0,41** (IC95%: -0,06 a 2,77; I²: 61%); para mialgia, a OR foi de **0,74** (IC95%: -0,22 a 2,46; I²: 1%); e para ascite, a OR foi de **0,84** (IC95%: -0,48 a 1,48; I²: 0%).

**Conclusão:**

O uso de estatinas está associado à melhora da sobrevida em pacientes com cirrose hepática. No entanto, não conseguimos determinar a razão desse efeito benéfico.

## INTRODUCTION

Liver cirrhosis and its complications, such as portal hypertension (PH) and hepatocellular carcinoma (HCC), are associated with high morbidity and mortality rates worldwide^1^. It is a significant public health issue, ranking as the 12th leading cause of death in the United States^2^. Among the primary causes of cirrhosis, metabolic dysfunction-associated steatotic liver disease (MASLD), alcohol-related liver disease, and viral hepatitis are particularly notable. MASLD has become highly prevalent worldwide, posing a significant etiology of chronic liver disease and a public health challenge^2^ ^,^ ^3^.

The development of cirrhosis involves several mechanisms of liver injury, including necroinflammation and activation of hepatic stellate cells, leading to fibrogenesis and angiogenesis. These processes result in a diffuse disorganization of the hepatic architecture, ultimately leading to parenchymal extinction and morphofunctional rearrangement of the hepatic microvascular bed. This distortion increases resistance to portal venous flow, resulting in portal hypertension. Decompensated cirrhosis is the final stage of chronic liver disease, often leading to death unless a liver transplant is performed^4^.

According to BAVENO VII guidelines^5^, portal hypertension is defined as a hepatic vein pressure gradient (HVPG) greater than five mmHg, while clinically significant portal hypertension (CSPH) occurs when this gradient is greater than or equal to 10 mmHg. Liver cirrhosis is the primary cause of portal hypertension^6^. The ideal therapy for treating portal hypertension should aim to reduce portal pressure, decrease hepatic vascular resistance, and increase blood flow to the liver. Currently, beta-blockers such as propranolol, nadolol, or carvedilol effectively elicit the target hemodynamic response in less than half of patients, with approximately 30% of patients having some contraindication to their use^6^ ^,^ ^7^.

Statins, which are lipid-lowering medications, work by competitively inhibiting 3-hydroxy-3-methylglutaryl-coenzyme A (HMG-CoA) reductase, a key enzyme in cholesterol synthesis. This inhibition prevents the conversion of HMG-CoA to mevalonate, a precursor of cholesterol, ultimately reducing cholesterol biosynthesis^8^. Statins are primarily indicated for the treatment of dyslipidemias and provide well-documented benefits for primary and secondary prevention of cardiovascular events. Physicians frequently prescribe them with caution in patients with chronic liver disease (CLD) due to their potential for hepatotoxicity. However, recent evidence suggests that hepatotoxicity is uncommon and that the risks of not using statins often outweigh the associated risks. Thus, statin therapy indeed appears to be linked to transient liver enzyme elevation rather than severe liver damage^6^ ^,^ ^9^. Moreover, recent findings indicate that statins may have a beneficial effect on cirrhosis due to their pleiotropic properties. Beyond reducing lipoprotein levels, statins enhance endothelial function, upregulate nitric oxide synthase, and reduce systemic inflammation, exhibiting antioxidant, antifibrotic, and anti-infectious effects^9^. The hepatic fibrogenic process primarily involves stellate cells, which store vitamin A and produce growth factors, cytokines, prostaglandins, and other bioactive substances^10^. Preclinical studies-both in vitro and in vivo-have demonstrated that statins can upregulate Kruppel-like factor 2 (KLF2), which induces the deactivation of hepatic stellate cells, resulting in reduced fibrosis. Additionally, statins promote endothelial production of nitric oxide, which facilitates vasodilation and reduces intrahepatic vascular resistance^6^ ^,^ ^11^. The pharmacological properties of various statin subclasses differ based on their activity on the HMG-CoA reductase enzyme, oral absorption, bioavailability, and hepatic metabolism. Simvastatin, atorvastatin, lovastatin, fluvastatin, and pitavastatin are lipophilic and metabolized by cytochrome enzymes, resulting in higher hepatic concentrations. Pravastatin and rosuvastatin are hydrophilic and experience minimal hepatic metabolism^9^. However, the clinical significance of these differences remains uncertain. In recent years, several clinical studies-primarily cross-sectional or retrospective epidemiological investigations-have reported the benefits of statin use in liver diseases, including primary biliary cirrhosis, chronic hepatitis C, chronic hepatitis B, metabolic associated steatotic liver disease, cirrhosis, and hepatocellular carcinoma^9^. Therefore, statins are emerging as a potential therapeutic option for managing the progression of liver cirrhosis and its complications, representing a significant advancement in treatment. The main objective of this meta-analysis was to evaluate the impact of statin use on number of patients with chronic liver disease alive at the end of the trials. We also aimed to determine the effect of statin use on the incidence of hepatic decompensation, which includes conditions such as portal hypertension, ascites, hepatic encephalopathy, variceal hemorrhage, and hepatocellular carcinoma.

## METHODS

### Eligibility criteria

We only included randomized clinical trials (RCTs) that met the following inclusion criteria: studies involving adult and elderly patients diagnosed with chronic liver disease, specifically liver fibrosis or cirrhosis of any cause, that evaluated exposure to statins and analyzed outcomes related to liver decompensation, prognosis, and survival.

### Search strategy

Data collection was conducted through the following databases: MEDLINE, EMBASE and SCOPUS. Only articles published in English were included. The search strategy was formulated using terms derived from Medical Subject Headings (MeSH) and Health Sciences Descriptors (DECS). To ensure a comprehensive search, we also reviewed the references of relevant studies identified during this process. This protocol adhered to the standardized items outlined in the PRISMA Protocol. The search terms focused on the population, the intervention, and the potential outcomes. Articles were selected up until June 1, 2024. The following search strategies were employed for the databases: - **MEDLINE/PubMed**: ((“Liver Cirrhosis”[mesh] OR “liver fibrosis”[tw] OR Cirrho*[ti] OR “chronic liver disease”[tw]) AND (statin*[tw] OR “Hydroxymethylglutaryl-CoA Reductase Inhibitors”[Mesh] OR “Cholesterol-lowering Drug” OR atorvastatin[tw] OR simvastatin[tw] OR rosuvastatin[tw])). - **EMBASE**: (‘liver cirrhosis’ OR ‘liver fibrosis’ OR ‘chronic liver disease’) AND ‘hydroxymethylglutaryl coenzyme A reductase inhibitor’ AND ‘randomized controlled trial’. - **SCOPUS**: (TITLE-ABS-KEY (cirrhosis) OR TITLE-ABS-KEY (liver AND fibrosis) AND TITLE-ABS-KEY (cholesterol-lowering AND drugs) OR TITLE-ABS-KEY (statins) AND TITLE-ABS-KEY (randomized AND controlled AND trial) OR TITLE-ABS-KEY (RCT)).

### Study selection and data extraction

The first evaluator performed the searches and imported those articles to the Rayyan-Intelligent Systematic Review platform, a free-to-use system sponsored by the Qatar Foundation that allows a rapid initial screening due to its several features available. Two independent evaluators performed a selection of articles to be read based on title and abstract, identifying studies that provided a complete reading, and excluding those not relevant to the present paper. If divergences had arisen, the evaluators would demand the opinion of a third evaluator. After that, the approved articles were listed in an Excel spreadsheet to collect data concerning the country of publication, number, gender, the average age of patients, number of subjects alive at the end of the trials, risk of bleeding due to esophageal varices, occurrence of hepatic encephalopathy, myalgia, ascites and reduction in portal pressure.

### Assessment of risk of bias and quality of evidence in studies

To evaluate the risk of bias, we used the Risk of Bias 2 (RoB 2) tool, and we employed the GRADE (Grading of Recommendations Assessment, Development, and Evaluation) scale to assess the quality of evidence. Two authors independently analyzed the papers, investigated the risk of bias, and evaluated the quality of evidence. In cases of disagreement between the two authors, a third author should resolve the issue.

### Statistical analysis

For our meta-analysis, we extracted the number of events and the total patient count to conduct meta-analysis of proportions using R software with the “meta” package (version 4.9-6), with results expressed in odds ratio (OR) and 95% confidence intervals (CIs). We combined the data using a random intercept logistic regression model, with adjustments made through logit transformation. For studies reporting zero events, we applied a continuity correction of 0.5. The Maximum Likelihood Estimator estimated variance between studies (tau²). The study assessed heterogeneity using the I² statistic, with classifications as follows: I² <40% indicates low heterogeneity; I² between 40% and 75%, substantial heterogeneity; and I² >75% indicates high heterogeneity.

### Ethical considerations

This study is a systematic review and meta-analysis. Thus, it did not require submission to a Research Ethics Committee (CEP). However, we registered this systematic review protocol in the International Prospective Register of Systematic Reviews (PROSPERO) on January 18, 2022 (registration number CRD42022298100).

## RESULTS

### Study selection

**FIGURE 1:**
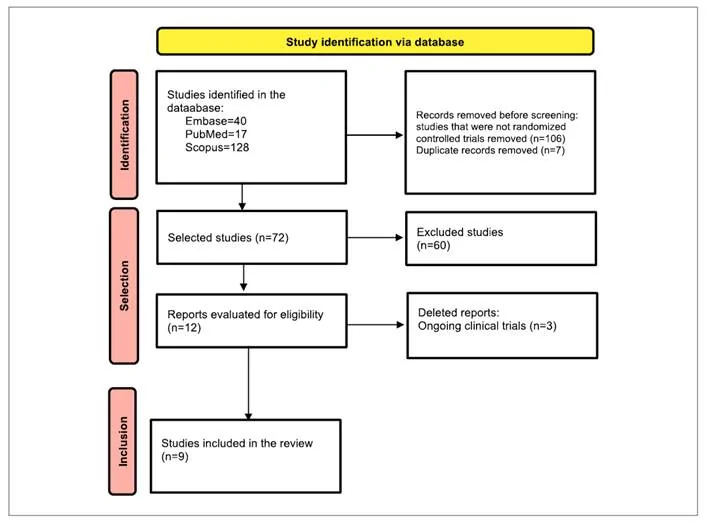
PRISMA diagram.

During the study selection process across various databases, we identified 648 articles, 79 of which were randomized clinical trials. We removed 8.8% of them due to being duplicates, resulting in 72 studies for further consideration. After reviewing the abstracts, we excluded 60 studies, leaving 12 that met the initial eligibility criteria. A detailed analysis of the inclusion and exclusion criteria ultimately led to the selection of 9 articles for qualitative analysis. Figure 1 summarizes the search strategy^12^.

### Characteristics of the studies

We reviewed nine randomized clinical trials involving a total of 811 cirrhotic patients with portal hypertension, with 401 patients assigned to the intervention group. The studies varied in duration, ranging from one month to two years. The research conducted by Elshazly et al. specifically focused on patients with cirrhosis caused by the hepatitis C virus (HCV), while the other studies included patients with various liver diseases. Table 1 summarizes the study characteristics^13^ ^-^ ^21^. Key outcomes measured in these trials included the occurrence of variceal gastrointestinal bleeding, worsening portal hypertension, changes in the hepatic venous pressure gradient (HVPG), survival rates, and mortality rates. Four studies (Abraldes et al., 2009; Bishnu et al., 2018; Pollo-Flores et al., 2015; Vijayaraghavan et al., 2020) reported a reduction in portal pressure, indicated by a decrease in HVPG, which led to improved hemodynamic patterns in patients. However, the study by Abraldes et al. (2016) found that statins did not reduce the risk of bleeding from esophageal varices, and Elwan et al. reported no protective benefit against new bleeding episodes. In the Abraldes et al. (2016) study, the authors randomized cirrhotic patients who presented within 10 days of variceal bleeding to receive either simvastatin with a beta-blocker or a placebo with a beta-blocker during a two-year follow-up. The results showed a significant survival benefit in the simvastatin group compared to the placebo group, with 17 patients (22%) in the placebo group dying compared to 6 patients (9%) in the simvastatin group (*P*=0.03). However, there was no significant reduction in rebleeding rates, with 22 patients (28%) in the placebo group experiencing rebleeding compared to 17 patients (25%) in the simvastatin group (*P*=0.583). The study by Vijayaraghavan et al. (2020) analyzed the addition of simvastatin to standard treatment with carvedilol in patients with portal hypertension. In the carvedilol monotherapy group, the mean reduction in HVPG was 2.95 mm Hg (17.3%), while in the carvedilol plus simvastatin group, it was 3.15 mm Hg (17.8%). The difference was not statistically significant (*P*=0.98). After three months, four patients experienced their first episode of variceal bleeding: two from the carvedilol group (2.4%) and two from the carvedilol plus simvastatin group (2.5%) (*P*=0.99). Therefore, adding simvastatin to carvedilol did not provide additional hemodynamic benefits for primary prophylaxis of variceal bleeding in cirrhotic patients. The study by Abd-Elsalam et al. (2018) evaluated 40 cirrhotic patients diagnosed with portal hypertension, with 20 assigned to a control group and 20 receiving simvastatin (20 mg for two weeks, followed by 40 mg for another two weeks). The authors utilized Doppler ultrasound to assess the effects of simvastatin on portal hypertension. They analyzed the portal vein diameter (PVD), portal vein velocity (PVV), portal vein blood flow (PVBF), and various indices related to hepatic and splenic artery resistance. The results indicated a significant decrease in the hepatic artery resistance index (HARI) after 30 days in the simvastatin group, while the study detected no changes in the control group (*P*<0.001). They concluded that simvastatin significantly decreased the portal hypertension index (PHI) (*P*<0.024) and the hepatic artery resistance index (HARI) in patients with cirrhosis and portal hypertension. Additionally, it improved hepatic perfusion, as evidenced by an increase in the modified liver vascular index (MLVI) (*P*<0.009). Table 2 summarizes the demographics of the patients included in the studies.

**TABLE 1:** Characteristics of included studies.

Study	Number of patients	Average age		Intervention		Duration time	Population studied	Outcomes of interest	Results
		Control	Statin	Control	Statin				
Abralde et al., (2009), Spain^13^	55	56±10	58±10	Placebo	Simvastatin 40mg/day	30 days	Cirrhotic patients with portal hypertension (HVPG ≥12mmHg)	Effects on HVPG, systemic hemodynamics and safety	The use of simvastatin decreased blood pressure portal pressure (29%) and improved hepatic perfusion.
	nC: 27								
	nE: 28								
Abraldes et al., (2016), Spain^14^	147	57.62±10.59	57.42±11.31	Placebo	Simvastatin 20mg/day for 2 weeks, followed by 40mg/day	2 years	Cirrhotic patient with portal hypertension after first episode of varicose UGIB	Rebleeding of esophageal varices	Simvastatin did not show superiority over placebo in reducing the rate of rebleeding or other hepatic decompensations.
	nC: 78								
	nE: 69								
Bishnu et al., (2018), India^15^	23	46.67±7.10	44±12.73	Proponolol 40mg/day	Propranolol 40mg/day + Atorvastatin 20mg/day	1 year	Cirrhotic patients with portal hypertension (excluded Child C)	Portal hypertension	Atorvastatin and propranolol achieved greater reduction in portal pressure than propranolol alone.
	nC: 12								
	nE: 11								
Elshazly et al., (2021), Egypt^16^	80	47.48±7.36	47.75±6.82	Placebo	Simvastatin 20mg/day for 2 weeks, followed by 40mg/day	1 year	Patients with HCV cirrhosis after first episode of variceal UGIB.	Survival in secondary prophylaxis of esophageal variceal bleeding.	Simvastatin may be associated with survival benefits in patients with Child A and B cirrhosis, with no difference in reducing the rate of rebleeding .
	nC: 40								
	nE: 40								
Elwan et al., (2018), Egypt^17^	40	50.8±6.993	51.5±6.692	Placebo	Simvastatin 20mg/day for 2 weeks and 40mg/day for 2 weeks	1 month	Cirrhotic patients with portal hypertension	Assess portal hypertension by Doppler ultrasonography	Simvastatin has been associated with reduced hepatic resistance without detrimental effects on systemic circulation.
	nC: 20								
	nE: 20								
Jha et al, (2019), India^18^	134	46.02±14.18	44.86±15.40	Carvedilol	Simvastatin 20mg/day for 1 week, followed by 40mg/day + Carvedilol	1 year	Cirrhotic patients after variceal hemorrhage	Esophageal variceal rebleeding and mortality	Adding simvastatin to standard treatment (carvedilol and rubber band ligation) may reduce rebleeding rate and improve survival for Child A and B patients.
	nC: 69								
	nE: 65								
Kronborg , et al. (2023), Denmark.^19^	78	63.5±8.6	59.0±10.14	Placebo	Atorvastatin 10mg/day for 4 weeks and 20mg/day for 20 weeks	6 months	Cirrhotic patients with portal hypertension (≥10 mmHg HVPG)	Mortality, Portal hypertension.	In patients with cirrhosis, atorvastatin is safe to use but does not reduce mortality, the risk of liver-related complications, or HVPG.
	nC: 40								
	nE: 38								
Pollo-Flores et al., (2015), Brazil^20^	34	58.5±13.5	56.5±8.7	Placebo	Simvastatin 20mg/day for 2 weeks, followed by 40mg/day	3 months	Cirrhotic patients with portal hypertension (≥5mmHg HVPG)	Portal hypertension	The use of Simvastatin decreased the portal vein pressure by 55%.
	nC: 14								
	nE: 20								
Vijayaraghavan et al., (2020), India^21^	220	52.5±8.48	51.1±9.41	Carvedilol	Simvastatin 20mg/day for 2 weeks, followed by 40mg/day + Carvedilol	3 months	Cirrhotic patients with portal hypertension (HVPG >12mmHg and presence of esophageal varices)	Primary prophylaxis for esophageal variceal bleeding	The addition of simvastatin to carvedilol for 3 months for primary prophylaxis of variceal bleeding did not improve the hemodynamic response relative to carvedilol monotherapy .
	nC: 110								
	nE: 110								

**TABLE 2:** Demographics of patients included in the studies.

Study	Average age		Female (%)		Etiology of alcoholic disease (%)										Liver disease stratification (%)						MELD		HVPG (mmHg) 2	
					Alcoholic		HCV		HBV		MASLD		Others		Child A		Child B		Child C					
	Control	Statin	Control	Statin	Control	Statin	Control	Statin	Control	Statin	Control	Statin	Control	Statin	Control	Statin	Control	Statin	Control	Statin	Control	Statin	Control	Statin
Abralde et al., (2009), Spain	56±10	58±10	22.2	39.3	44.44	39.28	48.1	50	7.4	0	-	-	0	10.71	59.3	64.3	29.6	35.7	11.1	0	6.9±1.9	6.2±1.3	19.8±3.8	18.5±7.2
Abraldes et al., (2016), Spain	57.62±10.59	57.42±11.31	32.1	34.8	55	49	22.1	27.5	2.6	1.4	4	1	7	8	24	15	62	68	14	17	10.03±5.32	10.15±4.40	-	-
Bishnu et al., (2018), India	46.67±7.10	44 ±12.73	0	18.8	50	36.36	0	0	8.33	0	8.33	0	33.33	63.64	-	-	-	-	-	-	11	11	17.17±6.12	15.55±5.30
Elshazly et al., (2021), Egypt	47.48±7.36	47.75±6.82	45	25	-	-	-	-	-	-	-	-	-	-	20	22.5	52.5	47.5	27.5	30	-	-	-	-
Elwan et al., (2018), Egypt	50.8±6.993	51.5±6.692	20	50	-	-	-	-			-	-	-	-	5	15	45	60	50	25	-	-	-	-
Jha et al, (2019), India	46.02±14.18	44.86±15.40	26.1	30.8	36.23	26.15	4.34	9.23	33.33	29.23	-	-	40.57	35.38	13	9.2	62.4	70.8	24.6	20	16.49±5.75	15.04±6.46		
Kronborg , et al. (2023), Denmark.	63.5±8.6	59.0±10.14	40	44.7	85	78.9	-	-	2.5	0	2.5	7.9	-	-	45	31.6	50	55.3	5	13.2	10.0±6.2	12.5±6.16	15.0±5.36	16.3±5.15
Pollo-Flores et al., (2015), Brazil	58.5±13.5	56.5±8.7	50	43	-	-	-	-	-	-	-	-	-	-	70	57.1	25	35.7	5	7.1	10.5±7	10±1.5	13±6	13±5.5
Vijayaraghavan et al., (2020), India	52.5±8.48	51.1±9.41	21.8	24.1	35.5	40	11.8	5.5	9.1	6.4	38.2	43.6	5.4	4.5	43.6	33.6	38.2	50.9	18.2	15.5	13.9 ±4.0	14.4 ±3.96	17.86±3.52	17.59±3.53

### Risk of bias

**FIGURE 2:**
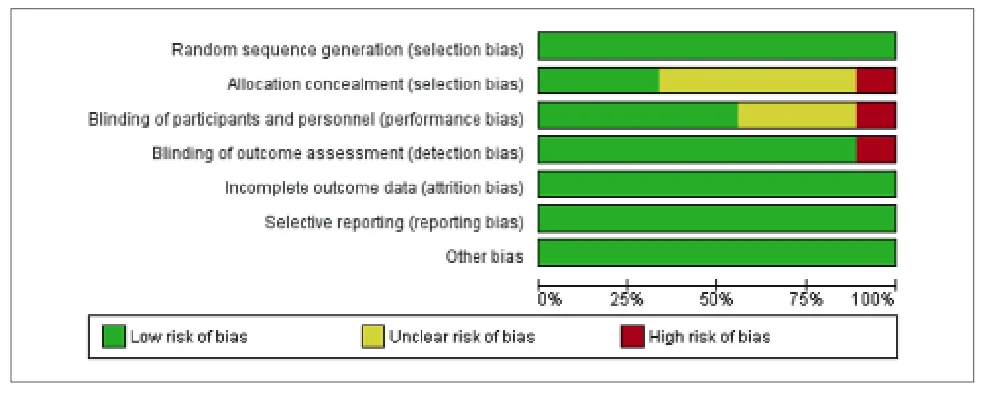
Risk of bias graph: review authors’ judgements about each risk of bias item presented as percentages across all included studies.

**FIGURE 3:**
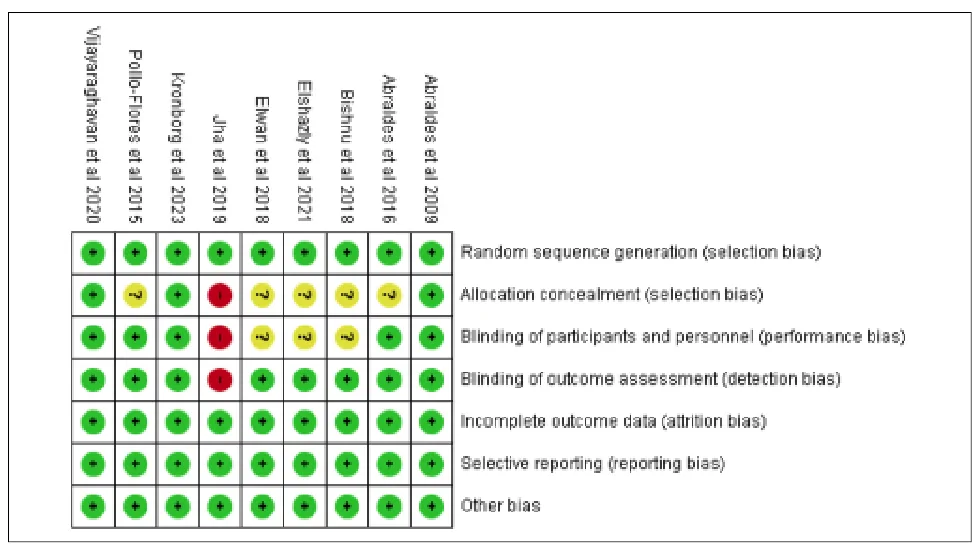
Risk of bias summary: review authors’ judgements about each risk of bias item for each included study.

We assessed the risk of bias using the ROB2 scale Figure 2. Most of the studies included in this meta-analysis have a low risk of bias, as shown in Figure 3. However, an article (Jha et al. 2019) presented a high risk in three of the variables analyzed: allocation concealment, blinding of participants and personnel, and blinding of outcome assessment.

### Patients alive at the end of the study

**FIGURE 4:**
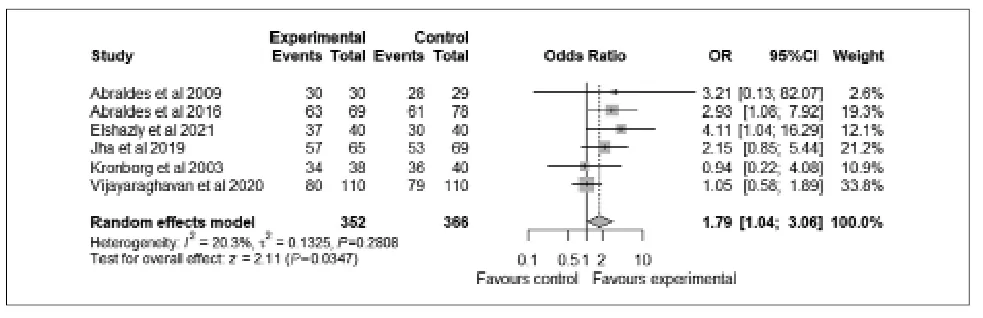
Forest plot - Patients alive at the end of the study.

Our meta-analysis included five papers in the analysis of patients alive at the end of the study after starting statin use, with a total of 718 patients (352 in the statin group and 366 in the control group). In total, 618 patients were alive, 301 in the intervention group and 287 in the control group. Overall, the OR between groups was 1.79 (95%CI, 1.04 to 3.06; I²: 20.3%), favored statin. Figure 4.

### Variceal gastrointestinal bleeding

**FIGURE 5:**
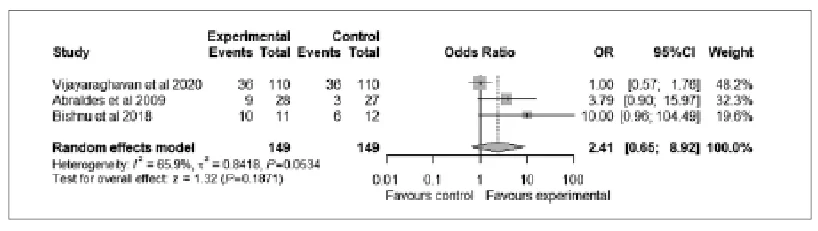
Forest plot - variceal gastrointestinal bleeding.

Five studies were included in the analysis of the variceal bleeding rate after the start of statin use, with a total of 604 patients (295 in the statin group; 309 in the control group). There were, in total, 87 events, 35 in the intervention group and 52 in the control group. Overall, the OR was 0.67 (95%CI, 0.41 to 1.09; I2: 0%) Figure 5.

### Hemodynamic response

**FIGURE 6:**
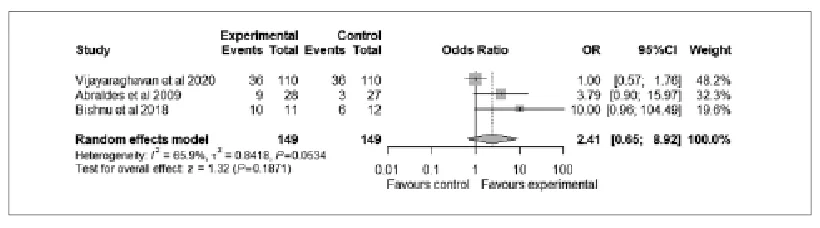
Forest plot - Hemodynamic response.

Our meta-analysis evaluated three papers comprising 298 patients (149 in the statin group and 149 in the control group); one hundred patients achieved hemodynamic response at the end of the study, 55 in the intervention group, and 45 in the control group. Overall, the OR was 2.41 (95%CI, 0.65 to 8.92; I2: 66%) Figure 6.

### Hepatic encephalopathy

**FIGURE 7:**
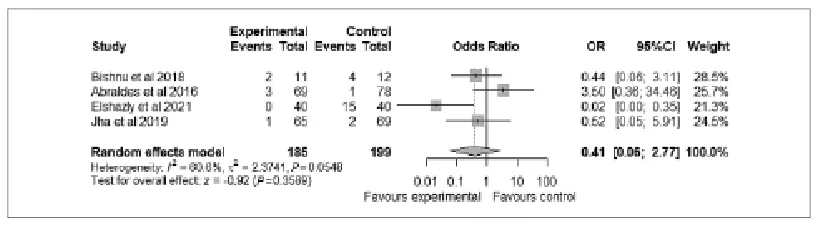
Forest plot - hepatic encephalopathy.

**FIGURE 8:**
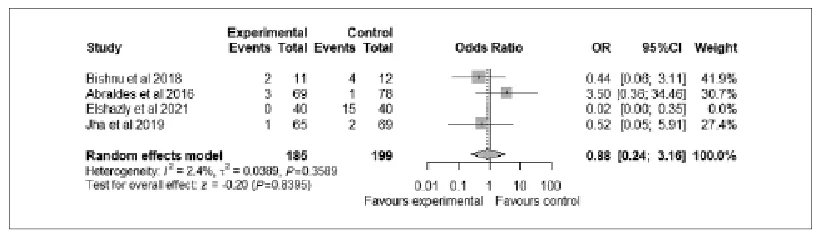
Forest plot - Hepatic encephalopathy (exclusion sensitivity analysis).

**FIGURE 9:**
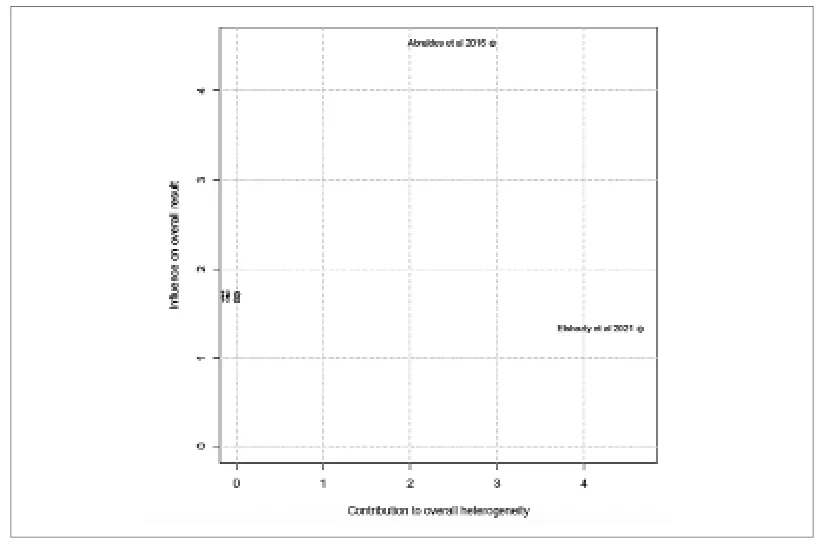
Baujat graph - Hepatic encephalopathy.

Regarding hepatic encephalopathy occurrence, our study analyzed four papers that included 384 patients (185 in the statin group and 199 in the control group). Of them, 28 patients presented hepatic encephalopathy, 6 in the intervention group and 22 in the control group. Overall, the OR was 0.41 (95%CI, 0.06 to 2.77; I2: 61%) Figure 7. Additionally, for this outcome, we performed an exclusion sensitivity analysis. After excluding the results of the Elsahzly et al. Figure 8 due to its heterogeneity, the OR was 0.88 (95%CI, 0.24 to 3.16; I2: 2%) Figure 9.

### Myalgia

**FIGURE 10:**
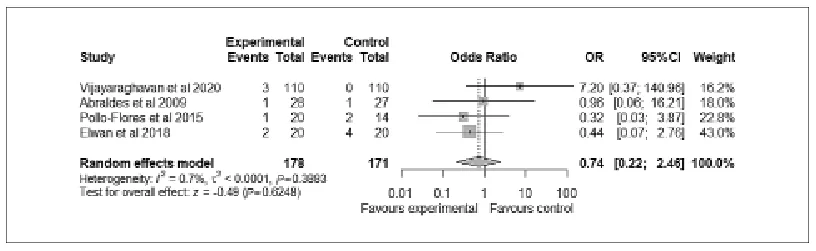
Forest plot - Myalgia.

Regarding myalgia, the meta-analysis evaluated four studies comprising 349 patients (178 in the statin group and 171 in the control group). Among these patients, 14 experienced myalgia as an adverse event, with 7 in the statin group and 7 in the control group. Overall, the odds ratio (OR) between groups was 0.74 (95%CI, 0.22 to 2.46; I²: 1%) Figure 10.

### Ascites

**FIGURE 11:**
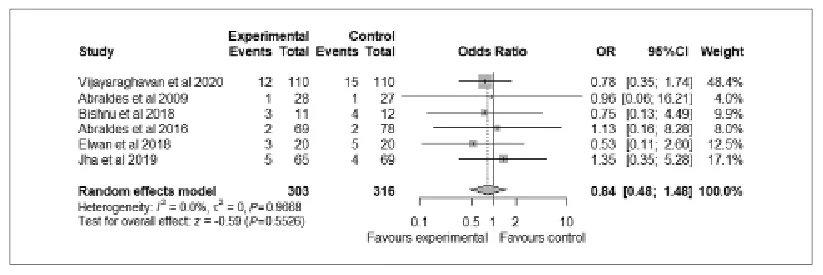
Forest plot - Ascites.

We included six papers, totaling 619 patients (303 in the statin group and 316 in the control group). Fifty-seven patients developed ascites, 26 in the intervention group and 31 in the control group. Overall, the OR was 0.84 (95%CI, 0.48 to 1.48; I2: 0%) Figure 11.

## DISCUSSION

In addition to their lipid-lowering properties, statins exhibit multiple pleiotropic effects, such as antioxidant, antiproliferative, and anti-inflammatory properties, as well as the ability to improve endothelial function and stimulate neoangiogenesis. Data suggests a potential benefit of statin for chronic liver disease. Observational studies of cirrhotic patients with cirrhosis suggested that statin use was associated with a reduced risk of hepatic decompensation, development of hepatocellular carcinoma, and death^22^. Statins should be particularly beneficial in MASLD and steatohepatitis patients, who often require the use of lipid-lowering agents^23^. Sharpton et al. reported a 40-60% lower risk of hepatic decompensation, a 25% lower risk of acute-on-chronic liver failure, a 30% lower risk of hospitalization for infection, up to a 20% reduction in the hepatic venous pressure gradient, a 10-30% reduction in all-cause mortality, and a 25-37% lower risk of HCC in patients who used statins^23^. 

Statins, generally, are well tolerated in patients with chronic liver disease, such as non-alcoholic fatty liver disease, primary biliary cirrhosis, and hepatitis C, and their use at appropriate doses can be of great help in the treatment of these patients. In their meta-analysis, Gu et. al.^24^ observed that statin treatment improved survival in patients with liver cirrhosis. Our study appeared to converge with their results; we evaluated the number of patients alive at the end of the trials and found that 85.5% of patients were alive in the intervention group compared to 78.4% in the control group [OR 1.79 (95%CI, 1.04 to 3.06]. Nevertheless, our paper failed to detect the reason for this benefit in the statin group. Although our meta-analysis found fewer complications related to liver decompensation in the intervention group, these results did not reach statistical significance. The incidence of ascites in the control group was 9.8%, compared to 8.6% among patients who used statins. Hepatic encephalopathy was detected in 3.24% of patients in the intervention group and 11.05% in the control group; hemodynamic response occurred in 36.9% of patients in the statin group and 30.2% of patients in the intervention group. We found that approximately 11.9% of cirrhotic patients undergoing statin use developed variceal gastrointestinal bleeding, while the control group had an average rate of 16.8%. On the other hand, Wan et al carried out a meta-analysis that demonstrated a statistically significant reduction in the rate of variceal hemorrhages among patients who used statins^25^.

Muñoz et. al.^22^ evaluated the safety of statins regarding muscle events in patients with cirrhosis and concluded that the safety profile of these drugs was excellent, especially with simvastatin. In our study, we found a similar incidence of myalgia in the control group (4.1%) and the intervention group (3.9%).

Studies using liver stiffness measurements (LSM) as an index of fibrosis have discerned that statin administration correlates with a reduced likelihood of advanced liver fibrosis^26^. Statin use has been shown to improve the prognosis of fibrosis due to decreased oxidative stress and inflammation in part related to effects on mesenchymal stem cell differentiation, endothelial function improvement, and hepatic vascular tone via nitric oxide synthesis and increases in endothelial progenitors, decreased stellate cell turnover, and protection against lipopolysaccharide-mediated damage and ischemia-reperfusion injury^23^. Izadpanah et al. hypothesized that inhibiting the mesenchymal stem cells (MSCs) potential to differentiate into macrophages would help to explain the statins’ pleiotropic effects. They treated MSCs with atorvastatin or pravastatin at clinically relevant concentrations and assessed their proliferation, differentiation potential, and gene expression profile. Both types of statins reduced the overall growth rate of MSCs. In particular, statins reduced the MSC’s potential to differentiate into macrophages, while they exhibited no direct effect on macrophage function^27^. Recently, Zhou XD et. al. analyzed the effects of statins on the long-term risk of all-cause mortality, liver-related clinical events (LREs), and liver stiffness progression in patients with MASLD. They collected data on patients with MASLD undergoing at least two vibration-controlled transient elastography examinations at 16 tertiary referral centers. They followed 7988 patients, with baseline LSM of 5.9 kPa (IQR 4.6-8.2), for a median of 4.6 years. At baseline, 40.5% of patients used statins, and cACLD was present in 17%. Statin was significantly associated with a lower risk of all-cause mortality (adjusted HR=0.233; 95%CI, 0.127 to 0.426) and LREs (adjusted HR=0.380; 95%CI, 0.268 to 0.539). Statin was also associated with lower liver stiffness progression rates in cACLD (HR=0.542; 95%CI, 0.389 to 0.755) and non-cACLD (adjusted HR=0.450; 95%CI, 0.342 to 0.592) but not with liver stiffness regression (adjusted HR=0.914; 95%CI, 0.778 to 1.074)^28^.

In contrast to the positive results from our study and the meta-analysis conducted by Gu et al. and Wan et al., a study published by Kezer et al. found that the use of statins did not provide a clinical benefit, at least within a six-month timeframe. However, the patients included in that study were either compensated or decompensated, which may have introduced variability in the study population and limited insights into the optimal timing for initiating statin therapy. 

In conclusion, our meta-analysis of randomized studies demonstrated that the use of statins was associated with a greater number of cirrhotic patients alive at the end of the trials compared to the control group. However, we were unable to determine the reason for this beneficial effect.

## Data Availability

Data-available-upon-request
