# ERRATUM: Role Of Statins In Cirrhotic Portal Hypertension: Meta-Analysis Of Randomized Studies

**DOI:** 10.1590/S0004-2803.24612026-E36

**Published:** 2026-10-05

 In article: Moreno MOA, Paz CLSL, Rodrigues MAN, Andrade ACD, Dezan MGF, Cavalcante LN, et al. Role Of Statins In Cirrhotic Portal Hypertension: Meta-Analysis Of Randomized Studies. Arq Gastroenterol. 2026;63:e25036. Doi:10.1590/S0004-2803.24612025-036. 

**Where it reads:**

 Our meta-analysis included **five papers** in the analysis of patients alive at the end of the study after starting statin use, with a total of 718 patients (352 in the statin group and 366 in the control group). In total, 618 patients were alive, 301 in the intervention group and 287 in the control group. Overall, the OR between groups was 1.79 (95%CI, 1.04 to 3.06; I²: 20.3%), favored statin. FIGURE 4. 

**It should read:**

 Our meta-analysis included **six papers** in the analysis of patients alive at the end of the study after starting statin use, with a total of 718 patients (352 in the statin group and 366 in the control group). In total, 618 patients were alive, 301 in the intervention group and 287 in the control group. Overall, the OR between groups was 1.79 (95%CI, 1.04 to 3.06; I²: 20.3%), favored statin. FIGURE 4.

